# AI-driven diagnosis of pneumococcal infections in children—a call for primary-care focused and a coherent evidence base

**DOI:** 10.3389/frai.2026.1866941

**Published:** 2026-09-21

**Authors:** Morro M. L. Touray, Mirella Longo, Aftab Ala, Joaquin M. Prada, Dembo Kanteh, Uma U. Onwuchekwa, Richard Green

**Affiliations:** 1 School of Medicine, Cardiff University, Cardiff, United Kingdom; 2 Kings College Hospital, London, United Kingdom; 3 Surrey Institute for People-Centred AI, University of Surrey, Guildford, United Kingdom; 4 Department of Comparative Biomedical Sciences, School of Veterinary Medicine, University of Surrey, Guildford, United Kingdom; 5 Strategic Services, MRC Unit The Gambia at London School of Hygiene and Tropical Medicine, Banjul, Gambia; 6 Centre for Vaccine Development-Mali, Ex-Institut Marchoux, Bamako, Mali; 7 Centre of Excellence on Ageing, School of Health Sciences, Faculty of Health and Medical Sciences, University of Surrey, Guildford, United Kingdom

**Keywords:** artificial intelligence-AI, children, diagnosis, economic evaluations, machine learning, paediatrics, pneumococcal infections, pneumonia

## Abstract

**Introduction:**

Despite widespread pneumococcal vaccination programmes, the disease remains a leading cause of paediatric morbidity and mortality globally. Persistent barriers, including limited vaccine access and coverage, cold-chain constraints, and delayed or inaccurate diagnosis, disproportionately affect rural communities, primary healthcare settings, and populations with low socioeconomic status. These same settings typically lack the radiological expertise required for timely diagnosis.

**Problem:**

Traditionally, diagnosis is done through chest X-ray interpretation by trained paediatricians or radiologists, who are rarely available in remote or resource-limited facilities. Artificial intelligence (AI)-aided diagnostic tools offer a potential pathway to address these gaps, enabling timely and accurate diagnosis, improved access, appropriate treatment, reduced human suffering, and potential cost-savings for healthcare providers and families.

**Evidence gap:**

To date, very few studies have evaluated AI-driven diagnostic tools specifically for pneumococcal infections focusing on children. Additionally, existing research is characterised by multiple shortcomings such as limited population diversity, small sample sizes, lack of external validation, absence of standardised reporting, and a hospital-centric focus that overlooks primary and community care and low-resource settings.

**Proposed approach:**

Given the many unanswered questions, we propose a transparent comprehensive systematic scoping review to identify and synthesise relevant existing and emerging body of literature in this field with a focus on children. The review will map the extent, range, and nature of available evidence, identify critical gaps, and assess the scope of validation and implementation research to date. It will also provide a framework for the economic analysis.

**Conclusion:**

Future research must move beyond isolated proof-of-concept studies toward a coherent, transparent, collaborative, and evidence-driven agenda. This agenda should include rigorous economic evaluations to fully understand resource implications for healthcare providers, health systems, and societies, particularly in low-resource settings where the potential impact of AI-driven diagnostics could be greatest.

## Introduction

Despite routine use of pneumococcal vaccinations in childhood immunisation programs around the world, invasive pneumococcal disease continues to be a leading cause of paediatric mortality worldwide (Whitney, 2017). For example, pneumonia alone accounts for an estimated 809,000 annual deaths in children worldwide (Wei, 2023, p. 158). This was from 725,000 deaths (in the under-5 year old population) including approximately 190,000 neonates in 2017 (Whitney, 2017) representing an increase of about 11.6%. This highlights persistent gaps in preventive measures, diagnosis and treatment, particularly in low- and middle-income countries. Challenges such as low vaccine coverage, cold chain inadequacies, appropriate treatment access and serotype replacement continue to hinder the control of the disease.

For those children that contract the disease, proper and timely diagnosis remains a major bottleneck. Traditionally, chest radiography is the primary imaging modality for suspected paediatric pneumonia (Florin and Gerber, 2020) infections. However, its interpretation requires specialised radiological expertise that is often unavailable at primary care levels and is worst in high-burden or low-resource settings. This results in incorrect, misclassified or delayed diagnoses and leading to missed timely treatment windows.

There is evidence that artificial intelligence (AI) and machine learning (ML), in particular deep learning architectures such as convolutional neural networks (CNNs), have demonstrated potential effectiveness and cost-savings in medical image analysis (Devi et al., 2023; Chakraborty et al., 2023). More broadly, AI-driven diagnostic and treatment approaches are increasingly being applied across infectious disease more generally, spanning diagnostic imaging, syndromic surveillance and antimicrobial decision-support (Srivastava et al., 2024); however, this broader is generally focused on adult populations. Despite the promising clinical and economic potentials, their application to diagnosis of pneumococcal infections in children remains underutilised and methodologically underdeveloped.

In this special position and call-to-action paper, we argue that there need to be a transparent, coordinated, and clinically grounded evidence base to guide responsible AI-based diagnostic tool innovation, validation, and implementation in paediatric pneumococcal treatment. It is essential that such efforts address paediatric and adult populations separately, given their distinct clinical needs and diagnostic considerations. We also argue that there need to be a thorough evaluation of its economic impact potentials to assess its cost-savings and cost-effectiveness using appropriate economic evaluation frameworks and methodologies with a particular focus on primary care and less-resource settings.

## Aim and scope of this paper

For clarity, we wish to explicitly state the nature and purpose of this paper. This is not itself a systematic or scoping review, and it does not report the results of a completed evidence synthesis. Rather, it is a position and call-to-action paper given the scarcity of publications on AI-driven diagnosis of pneumococcal infection in children. Our purpose is therefore to set out what can and should be done to build a coherent, clinically useful and cost-effective evidence base, particularly for children receiving primary care in low-resource settings. One of the concrete steps we propose, and have now begun as a new piece of work, is a comprehensive scoping review that will systematically and reproducibly map this literature. The themes discussed below are drawn from literature identified as part of search strategy development for this short-communication, and is undertaken to motivate and frame the case for that forthcoming review and reviews by other researchers in this important area. This work should be read as a structured special commentary to inform future research prioritisation and agenda setting.

## Emerging themes from the current landscape

While current evidence is scattered, there are some recurring patterns that are visible. The below themes, identified through literature search (an informal, non-systematic identification of studies known to the author team, not a systematic search with pre-specified criteria), underscore the gap between technical promise and clinical readiness. A full, reproducible, PRISMA-ScR-compliant mapping of this evidence base, focused specifically on children as is the subject of our planned systematic scoping review, is fundamental. The themes presented below should be understood as structured expert commentary intended to motivate that undertaking, rather than as findings from the review itself (see Table 1).

**Table 1:** The identified emerging themes in AI-driven diagnosis of pneumococcal infections in children.

Theme	Key observation	Specific evidence gaps/patterns	Example or consequence
Lack of pneumococcal specificity	Most AI and ML models target “pneumonia” broadly; while some distinguish bacterial from viral or fungal pathogens, very few specifically distinguish *Streptococcus pneumoniae* from other bacterial or atypical pathogens (Zubair, 2024; Rickard et al., 2025).	Models rarely use microbiologically confirmed pneumococcal cases as ground truth (dos Santos and Baeßler, 2018; Ginsburg and McCollum, 2023, p. 2). No publicly available model is explicitly trained to detect pneumococcal consolidation versus other lobar pneumonias.	A model may correctly identify “pneumonia” but fail to differentiate pneumococcal effusion from *Staphylococcus* or *Mycoplasma*, leading to inappropriate treatment or antibiotic choice.
Inconsistent performance reporting	Common metrics (accuracy, sensitivity, specificity) are reported, but calibration, confidence intervals, and external validation are frequently missing (Youssef et al., 2023; Faes et al., 2020).	Few studies report precision-recall curves or F1 scores (critical for imbalanced paediatric datasets). Calibration (agreement between predicted probability and observed frequency) almost never reported. Confidence intervals often omitted for sensitivity/specificity.	A model with 90% accuracy on a dataset with 5% pneumococcal prevalence may have very poor positive predictive value, yet this is rarely discussed.
Dataset limitations	Widespread reliance on small, single-centre, adult-dominant, or poorly labelled datasets limits generalisability.	Most paediatric pneumonia datasets contain < 1,000 images. Few datasets include microbiological or PCR confirmation of pneumococcus. Radiographs often lack standardised acquisition protocols (e.g., varying kVp, rotation, patient positioning). Adult models are frequently applied to children without re-training or domain adaptation.	A CNN trained on adult chest X-rays (e.g., NIH ChestX-ray14) performs poorly on paediatric images due to differences in thymic shadow, cardiac silhouette, and rib curvature.
Limited external validation	Even when internal validation is performed, external validation across different populations, devices, or geographies is rare (Youssef et al., 2023; Faes et al., 2020; Yang et al., 2022).	Most studies validate on a single held-out set from the same institution. Cross-population validation (e.g., training on high-income country data, testing on LMIC data) is almost absent. Temporal validation (e.g., training on 2015–2018 data, testing on 2019–2020 data) is rarely reported.	A model developed in a tertiary UK paediatric hospital may fail when applied to portable X-ray images from a rural clinic in The Gambia due to differences in image noise, patient positioning, and disease spectrum.
Poorly documented implementation barriers	Few studies address real-world deployment challenges: workflow integration, regulatory approval, clinician trust, ethics, or cost-effectiveness.	No published RCTs of AI for pneumococcal diagnosis in children (Grote, 2021; Genin and Grote, 2021; Han et al., 2023). User acceptance studies (clinicians, radiographers) are almost non-existent. Regulatory pathways for paediatric AI pneumonia models remain unclear. Ethical issues (e.g., algorithmic bias, informed consent in LMICs, accountability for misdiagnosis) rarely discussed (Sarkar et al., 2024; Weiner et al., 2024; Murphy et al., 2021).	An AI tool with high AUC may be rejected by clinicians because it does not provide interpretable heatmaps or explainable features, reducing trust in its recommendations. Acceptability and useability study therefore need to be embedded in any meaningful study.
Paediatric-specific anatomical and pathological differences	Children are not small adults; models trained on adult data fail to account for developmental changes in thoracic anatomy and disease presentation.	No model explicitly accounts for age-stratified differences (neonate, infant, toddler, school-age etc.…). Thymic shadow in young children can be mistaken for consolidation. Pneumococcal complications (e.g., empyema, pneumatocele) have different radiographic appearance in children (Cho et al., 2019; McCarthy et al., 1999; Ferrero, et al., 2010).	A model may falsely label normal thymic tissue as “pathological opacity” in an infant, leading to overdiagnosis and unnecessary antibiotics. Age-stratification therefore need to be carefully and strategically factored in the development and training of models and in their evaluation studies.
Lack of standardised ground truth labelling	Reference standards for model training vary widely, reducing comparability across studies.	Some studies use radiologist consensus without microbiological confirmation. Others use clinical diagnosis (fever + cough + tachypnoea) without imaging or laboratory confirmation. Very few use a composite reference standard (e.g., culture + PCR + radiology + clinical follow-up).	Two models claiming “90% accuracy” may be incomparable if one used radiologist labels and the other used microbiologically confirmed cases. A comprehensive, extensive and exhaustive confirmation approach has to be adopted.
Neglect of LMIC-specific constraints	Most AI development occurs in high-resource settings, with little adaptation to low-resource environments where pneumococcal burden is highest (Victor, 2025; Alami et al., 2020; Istasy et al., 2022).	Models rarely tested on portable or low-dose X-ray systems common in LMICs. Internet-dependent cloud deployment is infeasible in many settings; on-device or edge AI is underexplored. Cost-effectiveness analyses for LMIC deployment are absent (Arab and Moosa, 2025; Wu et al., 2025; Vithlani et al., 2023).	A state-of-the-art CNN requiring GPU-accelerated cloud inference is unusable in a district hospital with intermittent electricity and no internet connectivity.
Absence of clinical impact studies	No prospective trials evaluating patient-relevant outcomes (e.g., time to appropriate antibiotics, mortality, length of stay, antibiotic stewardship).	All published studies to date are retrospective or technical validation. No studies measure whether AI changes clinician behaviour or patient outcomes. No health economic evaluations of AI for paediatric pneumococcal diagnosis (Arab and Moosa, 2025; Wu et al., 2025; Vithlani et al., 2023).	Even a perfectly accurate AI model might not improve outcomes if it is ignored by clinicians, introduces delays, or leads to over-investigation or is only hospital centred in urban areas and not accessible to populations mostly affected by the disease.

To situate these themes against a concrete quantitative baseline: no pneumococcus-specific model has, to our knowledge, reported externally validated performance figures in children, so the closest available comparator is general (non-pneumococcus-specific) paediatric chest radiograph AI, and even here reported performance depends heavily on how a tool is validated. A systematic review of AI applied to paediatric chest radiographs found that most published tools were developed and validated primarily on adult data before being applied to children (Padash et al., 2022). In one directly relevant study, a commercial AI tool approved for adults and applied without modification to a cohort of 2,273 paediatric chest radiographs achieved an overall sensitivity of only 67.2% (specificity 91.1%, accuracy 87.5%); performance rose to 86.4% sensitivity (specificity 97.9%, accuracy 96.9%) only once cardiomegaly and children aged 2 years or younger were excluded, since age was itself a significant predictor of the tool’s errors (Shin et al., 2022). This illustrates, with real figures rather than an assumed range, both the potential of repurposing existing AI tools and the substantial performance degradation that can occur when a model is applied outside the population for which it was developed – a caution that applies with at least equal force to any future pneumococcus-specific tool.

A further practical constraint deserves explicit discussion: microbiological confirmation of pneumococcal aetiology by culture or Polymerase Chain Reaction (PCR), while desirable as a reference standard, is frequently unavailable, delayed, or cost-prohibitive in the rural, primary-care and community settings (particularly in LMICs) in which these tools are intended to be deployed, reflecting long-standing, well-documented constraints on laboratory diagnostic capacity in many low-resource health systems (Petti et al., 2006). Any future model development and validation work will therefore need pragmatic, tiered reference standards, for example combining radiological and clinical criteria with microbiological confirmation only where feasible, and explicitly reporting which standard was used, so that performance claims can be interpreted appropriately against the diagnostic reality of the target setting rather than against an idealised, resource-rich reference standard that many children in these settings may not have access to.

### Beyond the clinic wall - aligning AI diagnostics with community-based and primary care policy

While the preceding themes reveal technical and evidence gaps in AI and ML driven diagnosis for pneumococcal infections, an equally important but often overlooked dimension is how such diagnostic tools might fit into health system policy, not just in low-resource settings but in high-resource settings as well. For AI to move beyond academic validation and into meaningful impact, it has to be designed to have alignment with strategic policy directions. This includes the decentralisation of care into communities for example the “10 Year Health Plan for England: fit for the future” (NHS England, 2025) and primary care strengthening as the cornerstone of paediatric pneumonia infection management.

### The policy and strategy imperative - from hospital-centric to community-integrated care

In many LMICs, the default diagnostic pathway for suspected paediatric pneumonia relies on referrals to secondary or tertiary hospitals, which are often distant, difficult to access, under-resourced, and overcrowded. This model is neither an equitable nor an efficient method of care delivery. Both the WHO’s Integrated Management of Childhood Illness (Benguigui, 2001; Gera et al., 2016) strategy and the Global Action Plan for Pneumonia and Diarrhoea (Rudan et al., 2013; Qazi et al., 2015) have emphasised the mainstreaming of community-based case management through trained health extension workers or community health workers, primary care first-contact diagnosis, and reduced reliance on chest X-rays in peripheral settings due to its cost and excess radiation implications and the attendant expertise constraints.

AI-driven diagnostic tools for digital chest X-ray interpretation, as currently conceived, may end up being implemented under a hospital-centric model if it is deployed only at referral or specialised facilities. However, with a purposeful and deliberate policy / strategy alignment backed up with tailored training, the use of these tools can instead enable the decentralisation and community-based care agenda in several ways. See Table 2.

**Table 2:** Potential contributions of AI-based diagnostic tool to community- and primary-care systems.

Policy goal	Current barrier	Potential contributions
Community-based triage	Community Health Workers (CHWs) lack imaging expertise, necessary clinical training and cannot therefore interpret X-rays (Aderinto, 2023) or comfortably use such diagnostic systems.	Portable or handheld X-ray devices with on-device AI could allow CHWs to obtain and interpret images at village levels, with binary outputs (e.g., pneumococcal consolidation likely / unlikely). It can present training opportunities for CHWs in the various aspects of the system.
Strengthening primary care diagnosis	Primary health centres often have X-ray machines but no radiologist (Aderinto, 2023; Sekiguchi and Collens, 1995).	AI as a clinical decision support tool could provide immediate, standardised interpretation, reduce referral delays and enable same-day antibiotic initiation or appropriate treatment commencement. Can also provide opportunities for the training of primary healthcare staff, in particular nurses, in the various aspects of the system.
Reducing unnecessary referrals	Fear of missing pneumonia or the lack of diagnosis expertise leads to over-referral, crowding hospitals (Chambers et al., 2020; Muñoz et al., 2021, p. 2).	With high-negative-predictive-value, AI diagnostic tools could safely rule out pneumococcal pneumonia, allowing primary care clinicians to manage non-severe cases locally.
Targeted prevention and surveillance	Pneumococcal vaccination programmes lack real-time burden data at community level (Weeks et al., 2024, p. 9).	AI integrated with routine primary care imaging could generate population-level incidence maps, guiding vaccine campaigns and serotype surveillance.
Equitable access	Rural and remote populations have lowest access to diagnostic imaging (Sachdev et al., 2021; Arnold et al., 2021, p. 1).	Low-cost, ruggedised, solar-powered X-ray-AI systems could be deployed in mobile clinics or community health posts, reducing geographic inequity.

The care pathways for children with suspected pneumonia can be adapted when AI-driven technologies are implemented, specifically in the context of primary care and low-resource settings (Figure 1).

**Figure 1:**
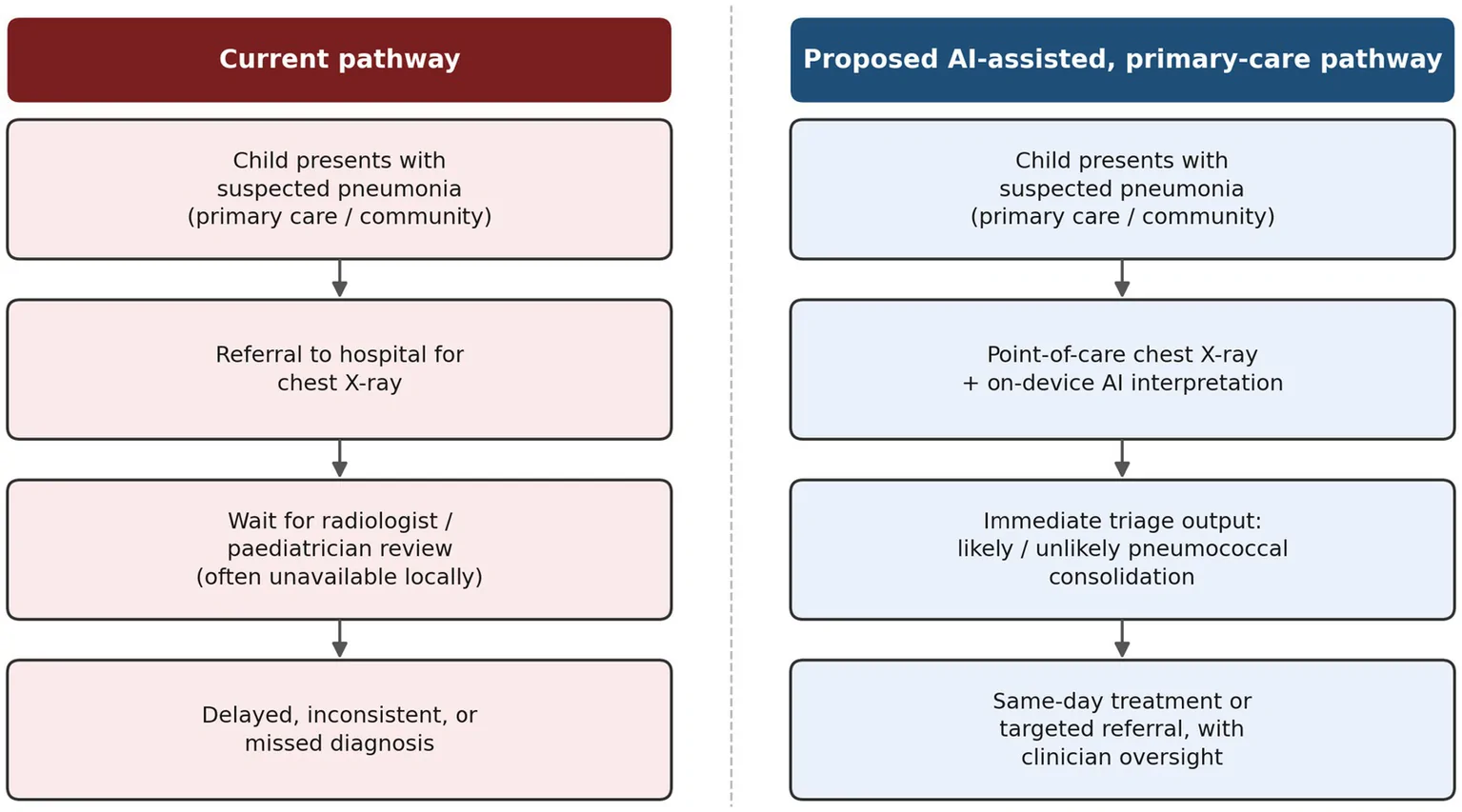
Schematic comparison of the current, hospital-dependent diagnostic pathway for paediatric pneumonia and the proposed AI-assisted, primary-care-based pathway. The AI-assisted pathway is illustrative and forward-looking: no component has yet been prospectively validated in the community settings shown.

Notwithstanding the potential befits and contributions of AI aided diagnostic tools for paediatric pneumococcal infections, as suggested in the previous sections, we believe that several questions must be answered. They include:

- *Randomised controlled trials*: Has efficacy been demonstrated in a randomised controlled trial comparing AI aided diagnosis with exclusively human interpretations (Grote, 2021; Genin and Grote, 2021; Han et al., 2023)?
- *Task shifting and scope of practice implications*: Can CHWs safely and legally use AI diagnostic tools? What training and supervision would be required?
- *Power infrastructure and connectivity*: How can AI function in settings with poor and intermittent power supply, no or poor internet connections, or limited cloud access?
- *Financing and costs*: Who would pay for the deployment at community levels and primary care settings? Have cost-effectiveness questions been answered, i.e., is the diagnostic tool more cost-effective than current care and referral pathways (Arab and Moosa, 2025; Wu et al., 2025; Vithlani et al., 2023)?
- *Referral and feedback loops*: How does a positive diagnosis of an AI aided diagnostic tool trigger appropriate referral, treatment, and follow-up? Without a proper and efficient integrated referral system and clear clinical guidance, infections may be identified but not necessarily change outcomes (Walcott-Bryant et al., 2020, p. 8).
- *Unintended consequences and equity*: Could low efficacy - false negatives at community level erode trust in CHWs? Could the deployment of the diagnostic tool increase disparities for example if it is initially placed only in better-resourced primary care centres (Victor, 2025; Alami et al., 2020; Istasy et al., 2022)?
- *Adequate policy engagement*: has there been proper and adequate engagement of policy makers, relevant healthcare professionals and stakeholders? All the relevant stakeholders need to be prospectively engaged to co-design regulatory, training, and financing frameworks before any large-scale deployment (Silvera-Tawil, 2024; van Gemert-Pijnen, 2022, p. 13).
- *Ethical considerations*: How ethical considerations can be adequately addressed - specifically transparent governance, robust model and safety validation, equitable access, and responsible data access, storage, and sharing?

## The promises and the gaps

In theory, AI-based chest X-ray interpretation of pneumococcal infections offers several advantages for primary care, high-burden and low-resource settings. These include enhanced diagnostic accuracy in the absence of radiological expertise, faster clinical decision-making in high-volume settings, standardised interpretation thereby reducing variability between readers, and the potentials for its scalability and access improvement.

Notwithstanding these potentials, a striking paradox exists - while AI-based diagnosis is rapidly expanding in other fields (Chakraborty et al., 2023; Jiang et al., 2022), research targeting pneumococcal-specific paediatric diagnosis remains sparse and under-utilised (Rickard et al., 2025; Togunwa et al., 2025). AI-based diagnostic tool would also face issues of broader pneumonia classification (pneumonia vs. no pneumonia), viral vs. bacterial infection differentiation and importantly adult or children or mixed age populations.

Very few seem to explicitly address pneumococcal infections in children (Rickard et al., 2025). Fewer even report detailed dataset characteristics, robust performance metrics, or external validation such as in randomised clinical trials (Genin and Grote, 2021; Han et al., 2023). These are important because pneumococcal disease has distinct radiographic features, e.g., lobar consolidation, parapneumonic effusions (McCarthy et al., 1999; Ferrero, et al., 2010). Applying generic models without robust and comprehensive pneumococcal-specific validation would risk misclassification, wrong diagnosis and inappropriate clinical management including unnecessary antibiotic prescribing. This highlights the need for a comprehensive structured systematic scoping review to identify knowledge gaps and/or inform further research and trials on the innovation.

## Why a scoping review is needed now

While a systematic review would be most preferable as it provides precise, evidence-based answers to inform clinical practice, policy, or further research, a systematic scoping review would be appropriate at this juncture as not much has been done in the area with very few publications and peer-reviewed articles. A scoping review will therefore provide a broad overview of research in the area, clarify concepts, and importantly identify knowledge gaps (Peters et al., 2021; Campbell et al., 2023; Khalil et al., 2020). A scoping review is also appropriate for the following reasons:

- Fragmented evidence hinders translation

Existing studies vary widely in terms of models’ architectures, dataset sizes and quality, confirmation standards (radiologist consensus, microbiological confirmation), how performance have been reported in terms of (accuracy, sensitivity, specificity, and calibration), and validation methodologies (internal only vs. external, cross-population). This heterogeneity would prevent meaningful comparisons and impede clinical readiness assessments.

- Paediatric datasets are critically scarce

Paediatric disease presentation, thoracic / chest anatomy, and radiographic findings fundamentally differ from those of adults (Padash et al., 2022). In that respect, models trained on adult datasets would therefore typically underperform in paediatric populations. Understanding current dataset availability and gaps, including their demographic diversity, image quality, and pneumococcal labelling, is a prerequisite for any meaningful progress in the development of an effective AI-based diagnostic tool.

- Implementation and equity dimensions are underexplored

Even high-performing models can face real-world hurdles such as, workflow integration with existing radiology and electronic health records systems (Kanbar et al., 2022; Nair et al., 2024; Magrabi et al., 2023), ethical considerations (Weiner et al., 2024; Murphy et al., 2021), equitable access and affordability in the sense of low-resource versus high-resource settings (Victor, 2025; Alami et al., 2020; Istasy et al., 2022), divergent regulatory approval pathways for AI-based medical devices, and clinician skills and interpretability. These issues are critical for any responsible deployment of such computer aided diagnostic tools.

- Direction for future research is needed

A scoping review can systematically map (Pollock et al., 2024) which AI and ML approaches have been applied to pneumococcal diagnosis and those focusing on children, what datasets exist and their quality and accessibility, how their performance has been reported and validated, and what and where evidence gaps exist. Such a map can guide developers, funders and researchers toward high- impact and priority intervention areas.

### The missing business case - why a comprehensive economic evaluation must accompany technical development

Even if AI aided diagnostics achieve high accuracy, and demonstrate benefits to primary- and community- care integration, a fundamental question will remain: Do they offer good value for money relative to current practice? As of now, economic evaluations involving comprehensive systematic assessment of costs and consequences has been almost entirely absent from the literature of computer aided pneumococcal diagnosis (van der Pol et al., 2021, p. 1412). This omission can be a critical barrier to the adoption by healthcare regulatory authorities and commissioners particularly in less resource settings.

Economic evaluations matter for several reasons. Healthcare budgets are severely constrained everywhere, and it is more acute in LMICs (Mackenzie et al., 2021, p. 4), who are largely donor dependent for their healthcare funding and where pneumococcal burden is highest (Adamu et al., 2021, p. 4). Every pound spent on the diagnostic tool must therefore be justified against alternative uses such as vaccines and vaccination infrastructure, antibiotics, or additional community health workers. Economic evaluations provide the necessary evidence base for such trade-offs. They can specifically answer:

- *Cost-effectiveness*: How much would the tool cost per additional correctly diagnosed case, per disability-adjusted life years averted, per quality adjusted life years, per infection prevented or per avoidable childhood death prevented (Arab and Moosa, 2025; Wu et al., 2025; Vithlani et al., 2023)?
- *Budget impact*: Should healthcare services adopt the tool and at scale, what would be the total financial implications, and which budget lines would be affected?
- *Cost-savings*: Can AI and ML diagnostic tools reduce healthcare expenditures by lowering unnecessary referrals, shortening hospital stays, or guiding more efficient treatment and antibiotic use (Yin et al., 2021; Angehrn et al., 2020, p. 11) or allow clinicians and clinical staff to concentrate on other serious cases?
- *The distributional effects*: Does AI-driven diagnosis reduce or widen inequalities in health and healthcare across socio-economic and ethnic groups, or geographic regions (Istasy et al., 2022; Alami et al., 2020)? This is particularly important if its use is concentrated in hospitals or referral facilities.

These questions should be appropriately answered even if there is a technically superior tool or else it may be underutilised, or worse, deployed inefficiently. AI and ML driven diagnostics generate potential pathways to cost-savings for paediatric pneumococcal infections for healthcare systems through various mechanisms (Table 3).

**Table 3:** Pathways to cost-savings.

Mechanism	Current practice	AI-Enabled pathway	Potential source of savings
Reduced unnecessary referrals, avoidable misdiagnosis and mistreatments.	Children with acute lower respiratory infections commonly undergo chest X-rays, often interpreted by radiologists, even when the illness is not pneumococcal (Porter et al., 2021, p. 13). Most children presenting suspected lower respiratory infections, in rural facilities LMICs, are given antibiotic treatments without proper investigations to ascertain cause of disease.	AI and ML aided tool at the primary care level can diagnose community-acquired pneumonia with a high negative percentage agreement, aiding in ruling out the condition (Porter et al., 2020, p. 5).	Avoided transport costs, hospital consultation fees, and family productivity losses. Clinicians can also concentrate on other serious cases.
Shorter hospital stays.	Delayed antibiotic administration is associated with increased length of hospital stay for children with community-acquired pneumonia, and uncertainty in diagnosis can lead to empiric broad-spectrum antibiotic use (Williams et al., 2013; Sandora et al., 2009, p. 1).	Rapid AI confirmation of pneumonia can lead to increased efficiency and performance in chest X-ray reading, potentially enabling earlier, more targeted interventions (Kwon et al., 2021, p. 2).	Reduced bed-days, nursing time, and hospital overhead.
Decreased reliance on scarce expertise.	Radiologist interpretation often requires tele-radiology, driven by staffing shortages and the need for after-hours coverage or remote expertise (Bashshur et al., 2016; Agrawal, 2022, p. 872).	Automated AI interpretation can be deployed as a point-of-care solution, particularly with digital and portable X-ray devices, providing instant classification and triage (Kharat et al., 2020).	Salary costs, travel subsidies, training and tele-radiology fees.
More efficient antibiotic use.	Overuse of broad-spectrum antibiotics, such as third-generation cephalosporins, is common in paediatric pneumonia due to diagnostic uncertainty and the challenges of differentiating between viral and bacterial causes (Morelli et al., 2023; Rossin et al., 2021, p. 2).	AI algorithms are being developed and studied for their potential to differentiate between viral and bacterial pneumonia, which would aid in pathogen differentiation Lhommet et al. (2019) Predicting the microbial cause of community-acquired pneumonia: can physicians or a data-driven method differentiate viral from bacterial pneumonia at patient presentation?; Lhommet et al. (2020) Predicting the microbial cause of community-acquired pneumonia: can physicians or a data-driven method differentiate viral from bacterial pneumonia at patient presentation?, p. 3.	Lower drug acquisition costs and reduced selective pressure for resistance (an indirect future saving).
Fewer follow-up visits.	Repeat chest radiographs are often used to monitor treatment response in children with pneumonia, although routine follow-up may not always be necessary if clinical improvement is observed (Virkki et al., 2005; Wacogne, 2003, p. 226).	Deep learning algorithms using serial chest radiographs can triage stable cases and detect interval changes during longitudinal follow-up, suggesting AI-assisted serial comparison for assessing resolution (Yun et al., 2023).	Reduced outpatient visits and repeat imaging costs.
Targeted vaccination surveillance.	Population-level vaccine impact is often measured through surveys and time-series analysis, which can be costly and subject to biases (Bruhn et al., 2016; Duarte et al., 2021, p. 233).	AI algorithms derived from chest X-rays can facilitate automated screening and provide quantitative analysis for pneumonia diagnosis and treatment monitoring, potentially extracting burden data from routine imaging (Kwon et al., 2021, p. 2).	Reduced survey / surveillance costs and more timely resource allocation

For any comprehensive economic evaluations, we propose a standardised framework that ensures comparability across studies and settings. As should always be the case in economic evaluations, the analysis should clearly and explicitly state the perspective. For LMIC policy decisions, the healthcare system perspective would be more relevant, but a societal perspective that accounts for household out-of-pocket costs, transportations for access, productivity loses, and other relevant opportunity costs should be adopted where possible.

WHO’s CHOICE data provides evidence-based data on health interventions to support priority setting, offering cost-effectiveness estimates, unit costs for health services, and disease models (Stenberg et al., 2018; Bertram et al., 2021, p. 2). The database utilises regional and country-specific data to guide decision-making on health investments (Johns et al., 2003; Stenberg et al., 2018, p. 1). CHOICE data is not regularly updated as such so inflation adjustments may have to be carried out (Bertram et al., 2017; Kumaranayake, 2000, p. 10). However, WHO also published WHO-INTEGRATE framework, which is a structured, six-criterion “evidence-to-decision” tool designed to help policymakers and guideline developers evaluate health interventions comprehensively (Norris et al., 2021; Movsisyan et al., 2024, p. 9). The framework incorporates WHO norms, such as human rights and equity, alongside health benefits, feasibility, and societal impact, ensuring transparent, context-specific decision-making (Murano et al., 2022; Movsisyan et al., 2024, p. 2). Table 4 provides key parameter inputs that must be factored in any meaningful comprehensive and relevant economic analysis of AI-based diagnostic tools.

**Table 4:** Parameter inputs for potential cost-savings assessments.

Parameter	Description	Data source
AI accuracy	Sensitivity, specificity, positive/negative predictive value at local disease prevalence, age-stratification, type of pneumococcal infection.	Scoping review / local validation study.
Current diagnostic pathway	Algorithms, referral rates, test use, treatment patterns.	Health facility surveys, routine data.
Unit costs	X-ray, AI software/license or software development, hardware, training, maintenance, clinician time, antibiotics, hospital bed-day, wages, overhead costs.	National health accounts, national reference costs, published international or national drugs prices, WHO-CHOICE, primary costing studies.
Health outcomes	Mortality (deaths averted), morbidity (infections prevented), sequelae (e.g., hearing loss, empyema).	Literature, vital registration, demographic surveillance.
Utilities / disability weights	For Disability Adjusted Life Years (DALYs) / Quality Adjusted Life Years (QALYs) calculations or other Health Related Quality of Life Measures.	Global Burden of Disease study, local preference elicitation.

## A call for coordinated, evidence-driven progress

To realise the potential of AI-aided tool for a paediatric pneumococcal diagnosis, the topic must move from isolated proof-of-concept studies to a coherent, collaborative, and evidence-driven research agenda. To achieve this, we are suggesting the following strategic priorities:

### Develop and share high-quality paediatric datasets

To have open, diverse, well-annotated datasets with microbiologically confirmed pneumococcal cases would be essential. However, data sharing must respect patient privacy and importantly local regulations. Standardisation improves transparency, reproducibility, and comparability.

### Prioritisation of external validation

Models must be tested across multiple and diverse populations, geographic settings, imaging systems, and clinical workflows (Youssef et al., 2023; Yang et al., 2022). Single-dataset accuracy may not necessarily be sufficient.

### Integration of clinical, ethical, and implementation science

AI aided diagnostic tool adoption would be a systems challenge, not solely a technical one. Multidisciplinary teams should include clinicians, ethicists, implementation scientists, and LMIC stakeholders from the outset (Weiner et al., 2024; Murphy et al., 2021).

### Evaluation of real-world impact

Prospective evaluations, including randomised controlled trials, cost-effectiveness analyses, and health equity assessments, would be fundamental to move from algorithmic promise to patient-centred practice, exploitation and eventual scaling up (Grote, 2021; Genin and Grote, 2021; Wu et al., 2025; Vithlani et al., 2023).

## Conclusion

There are healthcare transformative potentials for the use of AI in the diagnosis of pneumococcal infections in children. This is particularly profound in settings where radiological expertise is scarce. However, the current evidence base is fragmented, lacks pneumococcal specificity, and insufficiently addresses paediatric datasets, external validation, and implementation realities.

A rigorous systematic scoping review, such as the one currently being undertaking by our team, will provide a comprehensive map of this emerging field. It will identify critical gaps, synthesise available evidence, and guide future research and further clinical investigations. If an AI diagnostic tool is to meaningfully improve child health outcomes, especially in low-resource settings, the field must now shift from scattered innovation to coordinated, evidence-driven progress.

Such a tool should not be treated as inherently hospital centric. If aligned with decentralisation policies and primary care strengthening, it could become a transformative force for equitable, timely, and effective management of paediatric pneumococcal infections in the world’s most underserved settings.

The potential of the innovation will remain unrealised in practice unless accompanied by rigorous evidence of value for money. Healthcare authorities, stakeholders, donors and global health partners cannot make informed investment decisions without cost-effectiveness and budget impact analyses. The field must therefore move beyond accuracy metrics and usability studies to embrace robust economic evaluation as a core pillar of evidence generation. Only then can we determine whether AI represents not only a technological advance but also a wise use of scarce healthcare resources.

Even high-performing models can face real-world hurdles such as workflow integration with existing radiology and electronic health records systems, ethical considerations, equitable access and affordability in the sense of low-resource versus high-resource settings, divergent regulatory approval pathways for AI-based medical devices, and clinician skills and interpretability. These issues are critical for any responsible deployment of such computer aided diagnostic systems.
